# Respiratory effects of oral mitragynine and oxycodone in a rodent model

**DOI:** 10.1007/s00213-022-06244-z

**Published:** 2022-10-29

**Authors:** Jack E. Henningfield, Joseph V. Rodricks, Aaron M. Magnuson, Marilyn A. Huestis

**Affiliations:** 1 PinneyAssociates, 4800 Montgomery Lane, Suite 400, Bethesda, MD 20814 USA; 2 The Johns Hopkins University School of Medicine, Baltimore, USA; 3 Ramboll Health and Environment, Portland, USA; 4 Mountain West Research, LLC Dba CARE Research, Fort Collins, Portland, CO USA; 5 Thomas Jefferson University, Philadelphia, PA USA

**Keywords:** Kratom, Mitragynine, Oxycodone, Safety, Respiratory depression, Rat, Animal model, Pharmacokinetics, Pharmacodynamics, Opioid overdose epidemic

## Abstract

**Rationale:**

Kratom derives from *Mitragyna speciosa* (Korth.), a tropical tree in the genus *Mitragyna* (Rubiaceae) that also includes the coffee tree. Kratom leaf powders, tea-like decoctions, and commercial extracts are taken orally, primarily for health and well-being by millions of people globally. Others take kratom to eliminate opioid use for analgesia and manage opioid withdrawal and use disorder. There is debate over the possible respiratory depressant overdose risk of the primary active alkaloid, mitragynine, a partial μ-opioid receptor agonist, that does not signal through ß-arrestin, the primary opioid respiratory depressant pathway.

**Objectives:**

Compare the respiratory effects of oral mitragynine to oral oxycodone in rats with the study design previously published by US Food and Drug Administration (FDA) scientists for evaluating the respiratory effects of opioids (Xu et al., Toxicol Rep 7:188–197, 2020).

**Methods:**

Blood gases, observable signs, and mitragynine pharmacokinetics were assessed for 12 h after 20, 40, 80, 240, and 400 mg/kg oral mitragynine isolate and 6.75, 60, and 150 mg/kg oral oxycodone hydrochloride.

**Findings:**

Oxycodone administration produced significant dose-related respiratory depressant effects and pronounced sedation with one death each at 60 and 150 mg/kg. Mitragynine did not yield significant dose-related respiratory depressant or life-threatening effects. Sedative-like effects, milder than produced by oxycodone, were evident at the highest mitragynine dose. Maximum oxycodone and mitragynine plasma concentrations were dose related.

**Conclusions:**

Consistent with mitragynine’s pharmacology that includes partial µ-opioid receptor agonism with little recruitment of the respiratory depressant activating β-arrestin pathway, mitragynine produced no evidence of respiratory depression at doses many times higher than known to be taken by humans.

## Introduction

Kratom products are used globally for health and well-being, improved mood, sleep, attention, and coffee-caffeine-like alerting effects (Grundmann, 2017; Henningfield et al. 2018, 2022; Swogger et al. 2022). There is recreational use; however, many people who use opioids recreationally report kratom as a poor substitute for producing opioid-like euphoria but do find it useful to self-manage withdrawal and discontinue opioid use (Coe et al. 2019; Garcia-Romeu et al. 2020; Prozialeck et al. 2019; Swogger et al. 2022). Estimates of incidence and prevalence of intake vary from 2 to 16 million in the United States (US) (American Kratom Association 2019; Covvey et al. 2020; Henningfield et al. 2021; Palamar 2021; Schimmel et al. 2021) and many millions more globally, with the highest prevalence in Southeast Asia and Indonesia where kratom grows in abundance and has been widely consumed for centuries (Brown et al. 2017; Cinosi et al. 2015; Prozialeck et al. 2021; WHO ECDD 2021; Vicknasingam et al. 2010).

Deaths attributed directly to kratom use in the US and globally are rare and were not documented in Southeast Asia (Prozialeck et al. 2019; Ramanathan and McCurdy 2020). Analyses of kratom-associated deaths found that most of such deaths are more likely attributed to the use of other substances and causes not related to kratom (Gershman et al. 2019; Henningfield et al. 2019; National Institute on Drug Abuse 2020; Olsen et al. 2019).

Provisional estimates from the US Centers for Disease Control and Prevention (CDC) indicated that nearly 76,000 of more than 100,000 drug overdose deaths in 2021 were attributable to opioids (CDC 2021). In October 2021, the US Department of Health and Human Services (US DHHS) announced that drug overdose prevention efforts would include a substantial increase in harm reduction efforts to reduce opioid and other drug overdose deaths (US DHHS 2021). Kratom was not mentioned in the 2021 US DHHS announcement; however, US national surveys indicated that kratom is increasingly taken as a harm reduction strategy by thousands of opioid users to reduce and/or eliminate their opioid use related to pain and/or opioid use disorder (Grundmann 2017; Grundmann et al. 2018; Coe et al. 2019; Garcia-Romeu et al. 2020; Prozialeck et al. 2019, 2020, 2021; Swogger et al. 2022).

Oral mitragynine was investigated because mitragynine is the only alkaloid present at potentially biologically active levels in many widely marketed and consumed kratom products that also include mitragynine isolates. Many products have low or nondetectable concentrations of other alkaloids that may contribute to respiratory and other effects (Chakraborty et al. 2021; Sharma et al. 2019; Sharma and McCurdy 2021). Furthermore, unlike opioids and other substances widely used recreationally that are taken intravenously, insufflated, or inhaled (e.g., O'Brien 2015), kratom is taken almost exclusively by the oral route as dried kratom leaf powder teas, foods, or beverages to mask its bitter unpleasant taste, or leaf powder-filled capsules (Cinosi et al. 2015; Henningfield et al. 2018, 2022; Ramanathan and McCurdy 2020; Singh et al. 2016).

Earlier studies concluded that mitragynine has low respiratory depressant potential as compared to morphine or other µ-opioid agonists but did not include blood oxygen and related blood gas measures. Macko et al. (1972) measured respiratory rate in cats and dogs following morphine and/or codeine or mitragynine administration and found fewer effects from mitragynine. In another recently published study (Hill et al. 2022), 3 to 90 mg/kg mitragynine doses were compared to 3, 10, and 30 mg/kg morphine doses administered by oral gavage to mice tested in plethysmography chambers enabling measurement of minute respiratory volumes. Morphine produced dose-dependent decreases in minute volumes. The maximal effect of 90 mg/kg mitragynine was between that obtained with 10 to 30 mg/kg morphine.

The present study was designed to further evaluate mitragynine’s respiratory effects including effects on blood gases. The same basic approach as FDA utilized in its own studies comparing a broad range of substances to a prototypic respiratory depressing opioid, oxycodone (Xu et al. 2020, 2021) was employed. Oxycodone was selected by the FDA as the prototypic opioid for comparison and the dosing strategy was typical of what FDA often recommends to drug developers, namely inclusion of the therapeutic dose equivalent, and two or more supratherapeutic doses of oxycodone (6.75, 60, and 150 mg/kg).

The rational for selection of the mitragynine doses was to begin with a low dose utilized by other investigators (20 mg/kg), then systematically increase the dose until the highest tolerable dose or the highest chemically feasible dose due to ethical limitations followed by the laboratory on oral gavage (10.0 mL/kg or about 3.5 mL maximum) and limited mitragynine solubility. The laboratory determined that 400 mg/kg was the maximum dose based on solubility in Tween 20 solvent in a volume of approximately 3.5 mL (adjusted individually per animal based on actual body weight). The 400 mg/kg dose is substantially larger than the highest dose employed in most animal studies and is equivalent to 64.5 mg/kg in humans (dividing by 6.2 for allometric scaling). This is many times higher than is consumed by humans, whose typical per serving intake ranges from approximately 0.35 to 2.0 mg/kg per serving (Prozialeck et al. 2020; Singh et al. 2016; Swogger et al. 2022).

Respiratory effect measures were partial pressure carbon dioxide (pCO2), oxygen saturation (sO2), partial pressure oxygen (pO2), bicarbonate (HCO3), pH, and lactate in rat blood after oral mitragynine and oxycodone. Observable signs of drug effects for 12 h after dosing by veterinary-trained technicians with extensive experience in rat safety and pharmacokinetics studies are also reported (see details in the “Methods” section). The full pharmacokinetics of oxycodone and mitragynine will be reported elsewhere, whereas the present article reports maximum concentrations (*C*_max_) and time of *C*_max_ (*T*_max_), confirming dose-dependent increases in oxycodone and mitragynine.

## Methods

### Animals

Adult male Sprague Dawley rats (*n* = 48) approximately 80 days old weighed between 350 and 375 g at the time of surgical placement of carotid artery catheters at Charles River Laboratories. Actual body weights on dosing days ranged from 323.0 to 372.8 g. Animals were acclimated for 1 day at the CARE research facility due to concerns about the patency of the arterial catheters for collecting the primary blood gas and pharmacokinetic parameters. To ensure catheter patency, the catheters were flushed with heparinized saline every other day. A small amount of heparinized saline was left in the catheter as a “locking solution” to prevent clots from forming.

Rats were single-housed in a temperature-controlled room (20–26 °C) under a 12:12 h light/dark cycle, with 15–70% humidity and corn cob bedding. Animals had ad libitum access to Teklad Rodent Chow 2018 and filtered tap water, and no other medications were administered.

Animal care procedures were approved by the CARE Research Institutional Animal Care and Use Committee and animal welfare complied with the US Department of Agriculture’s (USDA) Animal Welfare Act (9 CFR Parts 1, 2, and 3), the Guide for the Care and Use of Laboratory Animals (National Academy of Sciences 2011), and laboratory standard operating procedures. Animals were euthanized and disposed of without necropsy in accordance with accepted American Veterinary Medical Association guidelines (Underwood and Anthony 2020).

### *Drug preparationand administration*

All drug doses were administered by oral gavage in maximum volumes of 10 mL/kg based on pre-session body weights for each animal following the ethical limitations of the laboratory. Table 1 includes doses and the number of days from delivery of the animals to Care Research from Charles River Labs that each dose was administered. Oxycodone hydrochloride from Sigma-Aldrich (Product Number O1378-500 mg) was dissolved in sterile water the morning of each test day. Doses were based on the weight of 100% pure oxycodone hydrochloride (6.75, 60.0, and 150.0 mg/kg). Mitragynine, 99.9% pure, extracted from Mitragyna Speciosa from Coryn Pharmaceuticals (Product Number 5057), was dissolved, or suspended in 20% Tween 20, because it is not water soluble. Mitragynine dissolved poorly in 20% Tween 20 above concentrations of roughly 10 to 15 mg/mL, requiring mitragynine doses to be prepared individually for each animal immediately before testing using vortex and sonication. To achieve the 240 and 400 mg/kg doses, the solution was in the form of a suspension, mixed well by vortex, and administered within 1 min of each preparation. Mitragynine was stored at room temperature in the dark and used within 62 days of receipt, with study completion within seven days from the first to the last dose.

**Table 1:** Administered doses, sequence of drug dosing, and the day following rat receipt the session was conducted. Test sessions were initiated between 7:30 and 8:30 AM during the light cycle

Group # drug dose mg/kg*	Sequence	Days after rats delivered for session
1. Oxycodone 6.75	5	8**
2. Oxycodone 60	4	4
3. Oxycodone 150	3	2
4. Mitragynine 20	1	1
5. Mitragynine 40	1	1
6. Mitragynine 80	2	2
7. Mitragynine 240	5	8**
8. Mitragynine 400	4	4

^*^Doses were calculated for each animal based on its pre-session body weight

^**^Blood gas analyzer failure required testing after a 4-day washout

The three initial mitragynine doses (20, 40, and 80 mg/kg) were the same as in earlier rat studies (Avery et al. 2019; de Moraes et al. 2009; Jagabalan et al. 2019), where data suggested maximum tolerable doses might be several times higher than 80 mg/kg. Drs. Henningfield and Huestis were briefed by the study director (Dr. Magnuson) 12 h after dosing each test day to determine if the next provisionally planned dose was likely tolerable and/or if it should be adjusted to document the dose–response curve as fully as possible. Because there were no observed behavioral effects, adverse events, or changes in blood gases at 80 mg/kg, the next dose was adjusted to 240 mg/kg, and the highest dose set to the maximum chemically feasible dose of 400 mg/kg.

### Test session sequence

Two groups were tested each day. Table 1 shows the dosing sequence and the days after the animals were delivered to the laboratory. Mitragynine doses increased from the lowest to highest doses, while oxycodone doses descended from the highest to lowest doses. Due to a malfunction of the blood gas analyzer, animals dosed with oxycodone 60 mg/kg and mitragynine 240 mg/kg had blood gases measured for only 2 h. No data from these doses were utilized, and the animals underwent a 4-day washout period. The oxycodone 60 mg/kg dose was administered the next day to naïve rats. Animals originally dosed with 60 mg/kg oxycodone were re-dosed with 6.75 mg/kg oxycodone after the 4-day washout, and animals originally dosed with 240 mg/kg mitragynine were re-dosed with 240 mg/kg mitragynine after the 96 h washout. Protocol changes were approved by the ethical committee and permitted rats adequate recovery from the few blood collections performed.

### Test session protocol

Animals were removed from their home cages for weighing at approximately 6 am each day and at 24 h post-dose, for drug administration and blood collections, and immediately returned to their home cages. Blood collections occurred pre-dose between 7:30 and 8:30 am and 1, 2, 4, 6, 8, and 12 h post-dose. A total of 300 µL whole blood (EDTA anticoagulant) was drawn at each time point (2.1 mL total). Approximately 100 µL was for blood gas analysis with a portable blood gas testing device (VetScan i-STAT Alinity V Handheld Analyzer with CG4 + cartridges (Zoetis/Abaxis/Abbott)) at 7 time points following procedures described by Xu et al. (2020). Blood gases were measured within 10 min after each blood collection.

A single blood gas measurement obtained data for all blood gas parameters at each time point for each animal and at each dose. A two-way ANOVA with multiple comparisons was performed, with the Dunnett test accounting for multiple comparisons, and significance set to *p* < 0.05. The remaining 200 µL blood was processed immediately for subsequent plasma oxycodone or mitragynine analyses, with storage at − 60 °C or below. Arterial ports were flushed with saline-heparin after each blood draw and every other day.

Observable signs were collected by the two veterinary-trained session monitors who identified and recorded adverse events and deviations from normal behavior. Observable signs included general activity (lethargy, impaired motor function, and righting response), response to stimulus (finger snap or clap near the animal), abnormal behaviors such as bracing (defined as a rigid posture with limbs slightly splayed), and apparent increases or decreases in respiration from normal/control. These items were given special attention as effects commonly observed in mu-agonist opioid studies, and our interest in comparing mitragynine’s effects to those of oxycodone, a prototypical mu-agonist opioid. Observations were conducted at each blood collection and monitored closely following dosing, and anytime an abnormality was observed by one of the two veterinary-trained session monitors. Observations were detailed in writing by the technician who made them. The technician observers were not blinded to treatments.

Six animals received each dose; 200 μL (K_2_EDTA) blood was processed to obtain plasma (~ 100 μL) for pharmacokinetic analysis. A total of 336 samples were analyzed for oxycodone and mitragynine in two separate validated LC–MS/MS assays. Pharmacokinetic analyses were performed with a non-compartment model (WinNonlin Phoenix professional, Version 8.3, Certara, Princeton, NJ) to determine pharmacokinetic parameters. The maximum plasma concentration (*C*_max_) and time of maximum plasma concentration (*T*_max_) following 6.75, 60, and 150 mg/kg oxycodone and 20, 40, 80, 240, and 400 mg/kg/d mitragynine group are presented. Due to space limitations and the many pharmacodynamic findings in this manuscript, a separate report will describe complete oxycodone and mitragynine pharmacokinetics.

## Results

### Oxycodone and mitragynine pharmacokinetics

Table 2 shows the maximum observed plasma concentrations (*C*_max_) for oxycodone and mitragynine. *C*_max_ increased by dose from 20 to 400 mg/kg, but each dose increase did not produce a significant increase based on a one-way ANOVA with Tukey’s post hoc analysis and a significance value of *p* < 0.05. Mitragynine *C*_max_ results were significantly different between the 20 and 40 mg/kg doses and the 240 and 400 mg/kg mitragynine doses, and the 80 mg/kg dose and the 400 mg/kg dose. Although the 240 mg/kg mean *C*_max_ was 5601 and the 400 mg/kg mean *C*_max_ was 7982 ng/mL, this increase was not statistically significant.

**Table 2:** Median maximum plasma concentrations (*C*_max_), with the minimum and maximum plasma concentration observed by drug and dose

Dose mg/kg	*n*	Median *C*_max_ ng/mL	Range ng/mL
Oxycodone			
6.75	6	33.5	10.7–51.5
60	6	39.9	34.5–1179
150	5*	131	93.2–5532
Mitragynine			
20	6	723	317–1010
40	6	1720	1043–2032
80	6	2497	1538–3125
240	4**	5182	3783–7444
400	6	7513	4404–10,096

^*^One animal’s data were not included in the analysis due to incomplete washout prior to redosing

^**^Another animal’s data were not included in the analysis due to incomplete washout prior to redosing and one eliminated due to extreme outlier for *C*_max_

The maximum plasma concentration generally occurred within 1–2 h (*T*_max_) but varied across animals, occurring as late as 12 h in some animals. Three animals were not included in the pharmacokinetic or pharmacodynamic analyses due to two animals not achieving concentrations below the limit of quantification (one in 150 mg/kg oxycodone group and one in the 240 mg/kg mitragynine group) after a 4-day washout period prior to redosing. One animal in the 240 mg/kg mitragynine group had a catheter blockage and tail blood was collected at time points 6, 8, and 12 h; however, for unexplained reasons, the blood analytic result was an extreme outlier for *C*_max_ at 23,425 ng/mL—a concentration threefold higher than any obtained with the 400 mg/kg dose. This result failed the outlier test and was not included in any analyses.

### Respiratory blood gas results

**Fig. 1:**
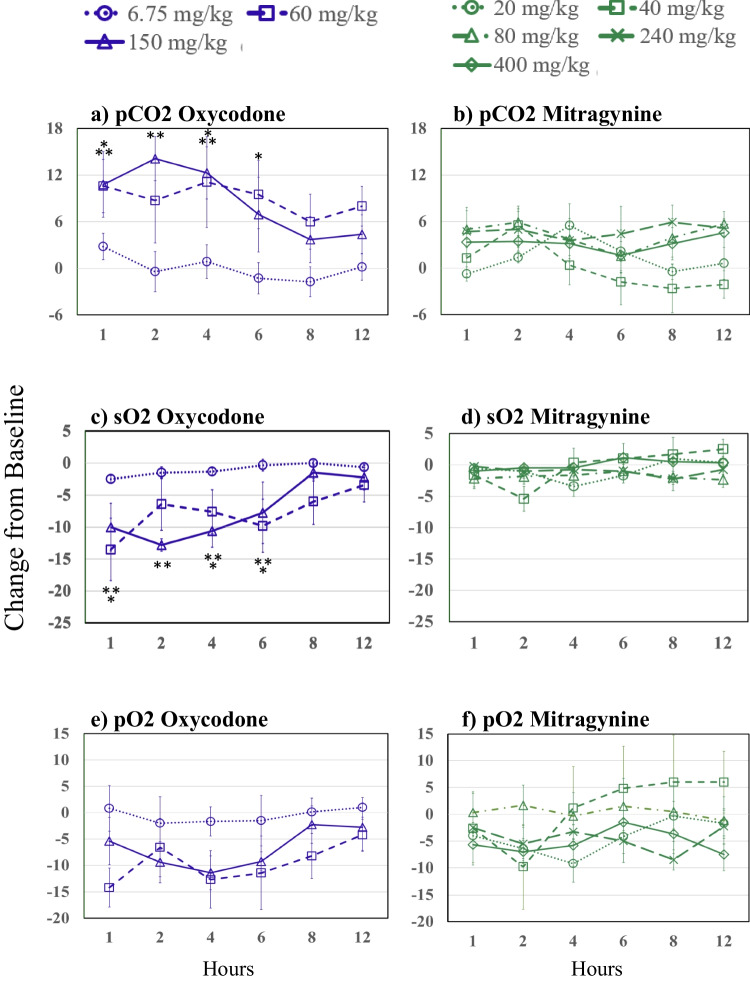
Changes from baseline over time following oxycodone in **a** partial pressure of carbon dioxide (pCO2), **c** oxygen saturation (sO2), and **e** partial pressure of oxygen (pO2); and changes from baseline over time following mitragynine **b** partial pressure of carbon dioxide (pCO2), **d** oxygen saturation (sO2), and **f** partial pressure of oxygen (pO2). Asterisks indicate significant differences from baseline (p≤0.05, one asterisks (*) 60 and two asterisks (**) 150 mg/kg oxycodone). Oxycodone is shown in purple. 6.75 mg/kg, dotted lines and circles; 60 mg/kg, dashed lines and squares; 150 mg/kg, solid lines and triangles. Mitragynine is shown in green; 20 mg/kg, dotted lines and circles; 40 mg/kg, short dashes and squares; 80 mg/kg, dashed and dotted lines and triangles; 240 mg/kg, long dashed lines and Xs; 400 mg/kg, solid lines and diamonds

Figure 1 presents the change from pre-dose baseline levels over time of oxycodone and mitragynine on the partial pressure of carbon dioxide (pCO2), which Xu et al. (2021) suggested is more sensitive than partial pressure of oxygen (pO2). None of the three blood gas parameters significantly deviated from baseline at the therapeutic 6.75 mg/kg oxycodone dose. The pCO2 was significantly increased relative to baseline at 1, 4, and 6 h for the 60 mg/kg oxycodone dose and at 1, 2, and 4 h for the 150 mg/kg oxycodone dose. There was a rapid decrease in sO2 and pO2 following the 60 and 150 mg/kg oxycodone doses, with the sO2 significantly depressed at 1, 4, and 6 h for the 60 mg/kg oxycodone dose and 1, 2, 4, and 6 h for the 150 mg/kg oxycodone dose, while no statistically significant decreases in pO2 were observed for either 60 or 150 mg/kg oxycodone. By 8 h post-drug administration, pCO2, sO2, and pO2 were no longer significantly different from baseline for higher oxycodone doses. There were no significant differences in pCO2, sO2, and pO2 following 20, 40, 80, 240, or 400 mg/kg mitragynine from baseline to 12 h.

**Fig. 2:**
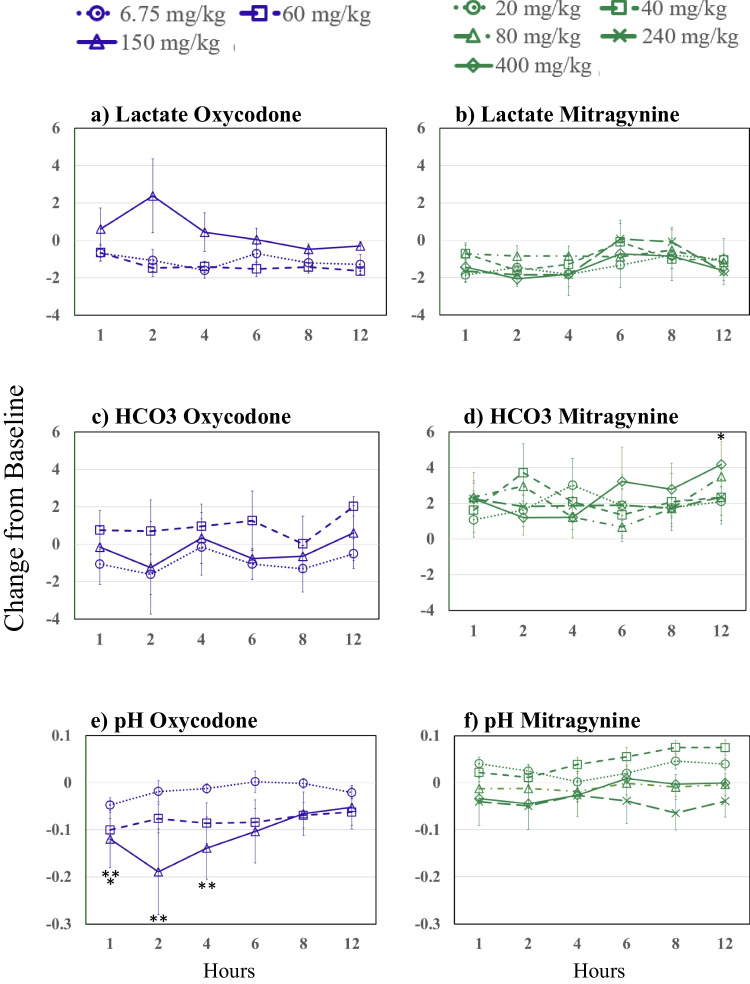
Changes from baseline over time and by dose (see key) following oxycodone on the left side panels (blue) and mitragynine on the right side panels (green) for lactate (top panels), bicarbonate (HCO3) (middle panels), and pH (bottom panels). Asterisks indicate significant differences from baseline (p≤0.05, one asterisk (*) 60 and two asterisks (**) 150 mg/kg oxycodone)

Figure 2 presents oxycodone and mitragynine effects on blood lactate, bicarbonate (HCO_3_), and pH. No mitragynine or oxycodone dose produced any statistically significant changes in HCO_3_ concentrations for 12 h after drug administration with the exception of the 400 mg/kg at 12 h post-drug administration. There were no significant changes from baseline in blood lactate following any oxycodone or mitragynine dose. Following 150 mg/kg oxycodone, the pH dropped significantly compared to baseline from 1–4 h and at 1 h after the 60 mg/kg oxycodone dose. There were no significant changes from baseline in pH after any mitragynine dose.

### Observable signs including potential drug-related adverse effects

There was no evidence of opioid or other drug effects or any behavioral observation in any of the animals receiving 6.75 mg/kg oxycodone or 20, 40, 80, and 240 mg/kg mitragynine that was visibly apparent to staff monitoring the animals.

Following 60 and 150 mg/kg oxycodone, the trained observers recorded numerous reports of behavioral effects including decreased activity, lethargy, apparently decreased respiration, and impairments to motor function. Four of the six animals at 60 mg/kg oxycodone showed overt signs of opioid exposure including pronounced behavioral effects and lethargy typical of opioids. One animal receiving 60 mg/kg oxycodone died between the 2 and 4 h time points, apparently as a result of the animal pulling its catheter and causing internal bleeding. These data were not used in the results. Four of the five remaining animals in the 60 mg/kg group appeared normal at 24 h, while one animal was found dead at 7:53 AM the next morning, almost 24 h following drug exposure.

The 150 mg/kg oxycodone dose produced notably stronger behavioral effects and lethargy typical of opioids with observable signs occurring in all 6 animals at 1–2 h. At 4 h post-dose, 3 of the 6 animals appeared normal. However, one of those animals was subsequently found dead approximately 5 h post-drug exposure. Two of the remaining 5 animals continued to exhibit behavioral signs at 6, 8, and 12 h, while all others returned to normal by 6 h. All 5 remaining animals appeared normal at 24 h.

The 400 mg/kg mitragynine dose produced milder observable signs than 60 and 150 mg/kg oxycodone. These observations included alteration of motor behavior, lethargy, and/or incoordination in 5 of 6 animals, with no observed abnormal behavior in the sixth animal. The slowed behavior suggested potentially decreased respiratory rate at 2 and 4 h in five animals.

Approximately 40 min after the 400 mg/kg mitragynine dose, one animal displayed “full body spasms” when picked up to be removed from the home cage for blood collection. Under observation (away from home cage) at approximately 1 h post-mitragynine dose, the animal exhibited what was described as a seizure-like activity for approximately 30 s that was described as moderate. No further seizure-like behavior was observed.

## Discussion

The purpose of this study was to evaluate the respiratory effects of kratom’s primary alkaloid, mitragynine, in comparison to oxycodone, a prototypic opioid that reliably produces dose-related respiratory depression and death in humans, rats, and other species. As far as we are aware, this is the first study of mitragynine’s respiratory effects that followed the approach published previously by Xu et al. (2020, 2021) that utilized blood gas assessment of respiratory function. The respiratory effects of a 20-fold range of mitragynine doses were compared to those of therapeutic and supratherapeutic doses of oxycodone for comparative assessments of the respiratory effects of drugs. The main finding was that mitragynine produced no evidence of respiratory depression at doses many times higher than typical human doses, whereas oxycodone produced the expected dose-related respiratory depressant effects consistent with its strong morphine-opioid (i.e., µ-opioid) receptor-mediated effects. These findings are consistent with mitragynine’s pharmacology that includes partial µ-opioid receptor agonism with little recruitment of the respiratory depressant activating β-arrestin pathway (Kruegel et al. 2016, 2019; Váradi et al. 2016).

Observable signs and blood pharmacokinetics confirmed that the administered doses produced the dose-related pharmacodynamic and pharmacokinetic results expected, namely, higher plasma concentrations and stronger behavioral effects.

The blood gas assessments revealed striking dose and drug-related effects. We found that oxycodone, in the same oral doses used by Xu et al. (2020), did not produce significant respiratory depressant effects at the therapeutic equivalent dose of 6.75 mg/kg, and had strongly depressant effects at 60 mg/kg and more severe and longer lasting effects at 150 mg/kg. In contrast, mitragynine did not produce dose-related respiratory depression or life-threatening effects at any dose, and there were no dose-related trends in blood oxygen or carbon dioxide. Consistent with the conclusion of Xu et al. (2021), pCO2 appeared to be a more sensitive measure of the respiratory depressant effects of oxycodone than blood oxygen measures; however, none of the blood gas measures showed dose-related changes associated with mitragynine administration.

This is not the first study to conclude that mitragynine does not produce opioid-like dose-dependent respiratory depressant effects. This was also demonstrated by Macko et al. (1972), and Hill et al. (2022) found no respiratory depressant effect at 3 mg/kg but did find some respiratory depression at 10 mg/kg, which they reported did not change at higher doses. Whether this difference from the present finding is unique to mice, measurement techniques or another factor is not known. In addition, Váradi et al. (2016, p. 7) reported that, in contrast to the dose-dependent respiratory depressant effects of morphine, a mitragynine analog “shows a lower propensity to cause respiratory depression and constipation compared with the canonical opioid, morphine.”

The human equivalent doses (HEDs) of those tested in this rat study are based on estimated body surface area dividing the rat doses by 6.2 (Nair and Jacob 2016). Based on this conversion, the HEDs of 20, 40, 80, 240, and 400 mg/kg are 3.2, 6.6, 12.9, 38.7, and 64.5 mg/kg respectively. Manufacturers of mitragynine-containing kratom extracts state that about 25 mg per serving or about 0.35 mg/kg in a 70 kg human is the minimal amount that provides sufficient self-reported benefits by consumers to support repeat sales. Some marketed products are labeled with this amount and commonly marketed products in Southeast Asia in the form of tea-like decoctions typically contain 22.5 to 75 mg but with wide variation across marketed products (Brown et al. 2017; Cinosi et al. 2015; Prozialeck et al. 2020; WHO ECDD 2021). Thus, it appears that the mitragynine doses administered in the present study range from the high end of consumer use (e.g., at the 20 mg/kg dose) to levels many times higher than would be voluntarily consumed or considered desirable or tolerable, regardless of their apparent limited effects on respiratory function. Some people who are self-treating chronic pain and/or using to maintain opioid abstinence may consume larger doses of 2–5 mg/kg 2–4 times per day (Cinosi et al. 2015; Figura et al. 2020; European Monitoring Centre 2015; McCurdy et al. 2020; Ramanathan and McCurdy 2020; Sharma and McCurdy 2021; Swogger et al. 2022; WHO ECDD 2021).

Self-report data posted on internet websites were summarized in several kratom use reviews (e.g., Cinosi et al 2015; Henningfield et al. 2018, 2022; Swogger and Walsh 2018; Veltri and Grundmann 2019). These data suggest that kratom intake is typically limited by non-life-threatening but discomfort-producing gastrointestinal symptoms and/or undesirable lethargy. Furthermore, for most kratom consumers, higher doses do not produce the powerful euphoria-like highs sought by people who recreationally use opioids, cocaine, and amphetamine and that is an incentive for the frequent dose escalation that contributes to the high risk of overdose deaths associated with such drugs (Swogger et al. 2022; Swogger and Walsh 2018; Henningfield et al. 2018, 2022).

An important caveat to the foregoing observations is that it should not be concluded that kratom is without life-threatening risks under some conditions and in some people, any more than such assumption should be made for any dietary or consumer product. It is possible, if not plausible, that in some yet-to-be documented combination with other drugs or underlying health conditions, high-dose kratom consumption could pose a serious risk. Therefore, it is prudent, as discussed elsewhere (e.g., Swogger et al. 2022) to not consume kratom with other drugs or in high dosages. For example, buprenorphine is a partial agonist that is approved for the treatment of pain and opioid use disorder and also produces little evidence of life-threatening depression over a broad range of doses. Buprenorphine did not produce life-threatening respiratory depression in humans even after a 16 mg intravenous dose (Umbricht et al. 2004; Huestis et al. 2013). However, buprenorphine is associated with overdose deaths when combined with benzodiazepines and other sedative-hypnotic drugs (Pergolizzi et al. 2015; Pergolizzi and Raffa 2019; Schuman-Olivier et al. 2013).

The absence of evidence for respiratory depressant effects in rats documented here does not rule out the possibility of respiratory depressant effects in some human conditions and in combination with other substances. Mitragynine in isolation and kratom produce diverse opioid and nonopioid mediated effects that warrant further study (Ahmad et al. 2022; Harun et al. 2022; Behnood-Rod et al. 2020; Hemby et al. 2019; Henningfield et al. 2018, 2022; Reeve et al. 2020; Sharma and McCurdy 2021; Wilson et al. 2020; Yue et al. 2018). However, as compared to opioids and other substances that contributed to the 2021 annualized rate of 108,000 drug overdose deaths, the relative risk of kratom appears to be far lower than that of opioids, methamphetamine and cocaine, alcohol, and many other substances of abuse (CDC 2021; Giroir 2018; Henningfield et al. 2019; United Nations Commission on Narcotic Drugs 2021).

The risk of kratom producing seizures is real but low and extensive surveillance to determine the conditions under which kratom may increase the risk may be necessary. For example, Boyer et al. (2008) reported a seizure in a person with no history of seizures following the ingestion of 100 mg modafinil combined with an unknown amount of kratom. Follow-up evaluation including computerized tomography and magnetic resonance brain imaging was normal. Modafinil itself can lower the seizure threshold and possibly cause seizures (Artsy et al. 2012; Bahramnjead et al. 2018). Thus, it is not known if kratom contributed but an interaction cannot be ruled out.

### Current findings in the context of recent advances in understanding the pharmacology of mitragynine

The finding that a broad range of mitragynine doses did not produce respiratory depression is not novel in itself because there are earlier studies of mitragynine across diverse doses and conditions, albeit at generally lower doses of 20–56 mg/kg in rats. These studies did not as intensively evaluate respiratory effects, and most did not include a pharmacokinetic assessment of mitragynine in comparison to a prototypic opioid. Several studies administered mitragynine to mice and dogs in experimental models with behaviorally functioning animals in conditioned place preference, self-administration, and intracranial self-stimulation models, reviewed recently by Henningfield et al. (2022), Ramanathan and McCurdy (2020), Sharma and McCurdy (2021), and WHO ECDD (2021). None of these studies reported life-threatening effects of mitragynine of 20–56 mg/kg and higher (e.g., Avery et al. 2019; Behnood-Rod et al. 2020; Maxwell et al. 2020; Obeng et al. 2021; Gutridge et al. 2020; Hassan et al. 2021; Kamble et al. 2021; Suhaimi et al. 2021; Todd et al. 2020). For example, a pharmacokinetics and safety study in beagle dogs reported a “mild transient sedation” lasting about 2–4 h after 5 mg/kg oral mitragynine but without clinically significant effects on vital signs (Maxwell et al. 2020). Váradi et al. (2016) found that morphine, but not a mitragynine analog, dose-dependently decreased respiratory rate in rats.

The main strengths of the present study include its dose-related comparison of oxycodone to substantially higher doses of mitragynine than were tested in most other studies and by close adherence to the protocol used in two published studies for evaluating respiratory effects (Xu et al. 2020, 2021). Its main limitation with respect to its goals was that the limit of tolerability, as defined by life-threatening effects, was not discovered and was limited by dosing restrictions. Future studies will consider alternative oral dosing approaches to enable the administration of higher doses. It is also possible that the two groups of animals that were not mitragynine naïve due to the blood gas analyzer failure were affected by their prior mitragynine exposure despite the 4-day washout period. Nor does this single ascending dose (SAD) approach address the effects of chronic dosing as can be informed by multiple ascending dose (MAD) studies. Future studies will help better understand the generalizability of the present findings, although the overall findings that the respiratory effects of mitragynine are weak albeit at lower doses as compared to morphine-like opioids are not novel. It also is important to evaluate the effects of mitragynine in combinations with drugs commonly taken with kratom including opioids, sedatives, alcohol, and stimulants. Further studies of mitragynine and other kratom constituents and analytes are clearly warranted to continue to advance science and public health.
